# SmartStep: A Fully Integrated, Low-Power Insole Monitor

**DOI:** 10.3390/electronics3020381

**Published:** 2014-06-18

**Authors:** Nagaraj Hegde, Edward Sazonov

**Affiliations:** Department of Electrical and Computer Engineering, the University of Alabama, 342 HM Comer, Tuscaloosa, AL 35487, USA

**Keywords:** Bluetooth low energy, sensors, low-power systems, flash memory management, Android

## Abstract

Shoe-mounted wearable sensors can be used in applications, such as activity monitoring, gait analysis, post-stroke rehabilitation, body weight measurements and energy expenditure studies. Such wearable sensors typically require the modification or alteration of the shoe, which is not typically feasible for large populations without the direct involvement of shoe manufacturers. This article presents an insole-based wearable sensor (SmartStep) that has its electronics fully embedded into a generic insole, which is usable with a large variety of shoes and, thus, resolves the need for shoe modification. The SmartStep is an always-on electronic device that comprises a 3D accelerometer, a 3D gyroscope and resistive pressure sensors implemented around a CC2540 system-on-chip with an 8051 processor core, Bluetooth low energy (BLE) connectivity and flash memory buffer. The SmartStep is wirelessly interfaced to an Android smart phone application with data logging and visualization capabilities. This article focuses on low-power implementation methods and on the method developed for reliable data buffering, alleviating intermittent connectivity resulting from the user leaving the vicinity of the smart phone. The conducted tests illustrate the power consumption for several possible usage scenarios and the reliability of the data retention method. The trade-off between the power consumption and supported functionality is discussed, demonstrating that SmartStep can be worn for more than two days between battery recharges. The results of the mechanical reliability test on the SmartStep indicate that the pressure sensors in the SmartStep tolerated prolonged human wear. The SmartStep system collected more than 98.5% of the sensor data, in real usage scenarios, having intermittent connectivity with the smart phone.

## 1. Introduction

Shoe-based activity and/or gait monitoring systems are gaining widespread popularity in research, as well as in the commercial market place. Shoe-based sensors are being used in applications ranging from studies of obesity to post-stroke rehabilitation, from energy expenditure studies to training activities in sports. One of the most popular applications for shoe-based sensors is gait analysis, and various analysis systems based on different sensor modalities have been proposed and applied both to healthy individuals and patients with neurological disorders [1–10]. In-shoe sensors have been applied for the monitoring of plantar pressure [11], ground reaction forces [12], estimation of center of mass displacement during walking [13], pedestrian navigation [14] and pedestrian tracking [15]. A number of publications describe the use of shoe-based sensor systems for biofeedback in rehabilitation applications, such as PDShoe [16], a system that provides biofeedback to improve the gait of Parkinson’s patients and that provides feedback on an Android smart phone [17]. Shoe sensors have also been applied in several different populations: children with cerebral palsy [18], Parkinson’s patients [4,16], post-stroke individuals [19,20] and, of course, healthy individuals [21].

Our laboratory developed various generations of a shoe-based platform named SmartShoe that incorporated pressure transducers and an accelerometer and that had been used in a number of different applications. SmartShoe had demonstrated the accurate (98%) classification of the six major postures and activities [21] in healthy individuals and the comparable recognition of postures and activities in post-stroke individuals [19]. The ability of SmartShoe to reliably recognize postures and differentiate between weight-bearing and non-weight-bearing activities enables accurate energy expenditure prediction [22] in models branched by activity. SmartShoe was also used for the estimation of the body weights of wearers [23] and to accurately capture the temporal gait parameters of healthy and post-stroke individuals [3].

Most of the above-mentioned shoe-based platforms, including the SmartShoes, have obvious limitations, mainly accommodating the sensors and wireless electronics in the shoes; either there is a need to modify the shoe or to attach additional hardware to the shoe. Such modifications are typically labor-intensive and may potentially diminish the original functionality of the shoe (e.g., by creating holes and allowing moisture into the shoe), while attachments may not be reliable and can create trip hazards. Commercially available sensor-equipped shoes that minimized such issues appeared as early as 1986 (PUMA RS100 Computer Shoe [24]) and continue to be manufactured to this day (Adidas 1 [25], Nike Lunar [26]). However, these commercially available shoe platforms do not offer all of the rich features that the research platforms offer (e.g., easy access to sensor data), which can be used in multiple studies and target applications.

Other important issues that concern in-shoe systems are the organization of low-power operation and wireless delivery of the sensor data. The average current consumption with the SmartShoe platform was 40 mA [27], and to use SmartShoes for one day of wear (12 h), a battery with a capacity of more than 480 mAh was needed. In-shoe systems designed for the long-term monitoring of gait or other parameters should present minimal burden, in terms of charging the shoe sensor batteries. In-shoe location is also problematic in terms of subject mobility, as the shoe data is typically delivered to a base station and the individuals cannot be always expected to be in the vicinity of the base station, such as a smart phone, used for data collection and logging. Finally, the monitoring system has to be robust enough for long wear.

In an effort to concisely address all the above-mentioned factors, we have developed SmartStep. SmartStep is a physical activity and gait monitor that is fully integrated into an insole, meets the low power requirements, addresses the issue of reliable data delivery in conditions of intermittent wireless connectivity and maximizes the convenience, applicability and social acceptance of the monitor. SmartStep is unobtrusive for the wearers, as the electronics, sensors, wireless module and rechargeable battery are encapsulated inside the insole. This helps in making the wearers less conscious of wearing an activity monitor while they perform their daily activities in free living conditions. In addition, SmartStep does not require the modification of the shoe, nor is there a need to attach additional hardware to the shoe. Compared to commercially available products, SmartStep offers rich features for the researchers interested in human subject studies of activity and gait. The device has data retention capability, and the individuals wearing the SmartStep need not be in the constant vicinity of the smart phones. With the use of Bluetooth low energy (BLE) along with all low-power electronic components, SmartStep consumes less power.

This article concentrates on addressing the following system design challenges:

- Managing low power in an “always on” wearable electronic system.
- Handling of the data retention scheme.
- Mechanical reliability of the wearable system for prolonged wear.

In addressing these challenges, this article describes the SmartStep hardware, firmware, Android application and specific experiments targeted at characterizing the power consumption, flash buffering scheme and reliability of the system.

## 2. Methods

### 2.1. SmartStep Hardware

We had reported a framework for the SmartStep development in our previous work [28]. This manuscript presents and characterizes the final prototype. The electronic printed circuit board (PCB) houses a low-power 3D accelerometer, ADXL346, a low-power 3D gyroscope, L3GD20, a low-power flash memory, MX25L16 (16 M bit), and Blueradios BLE module, BR-LE4.0-S2A, which is based on CC2540 SOC from Texas Instruments (TI). The PCB also has on-board power management circuitry and 3 pressure sensor interfaces. The accelerometer, gyroscope and Flash are all interfaced to the CC2540 on the same serial peripheral interface (SPI) bus with different chip select pins. The accelerometer and gyroscope both have the provisions to interrupt the CC2540 core. The design utilizes 16 out of 17 available I/O pins from the BLE module. The PCB is 24 mm × 19 mm in dimension and weighs 4 g. A rechargeable lithium battery, ML2020 (3 V, 45 mAh), powers the whole system.

**Figure 1.**
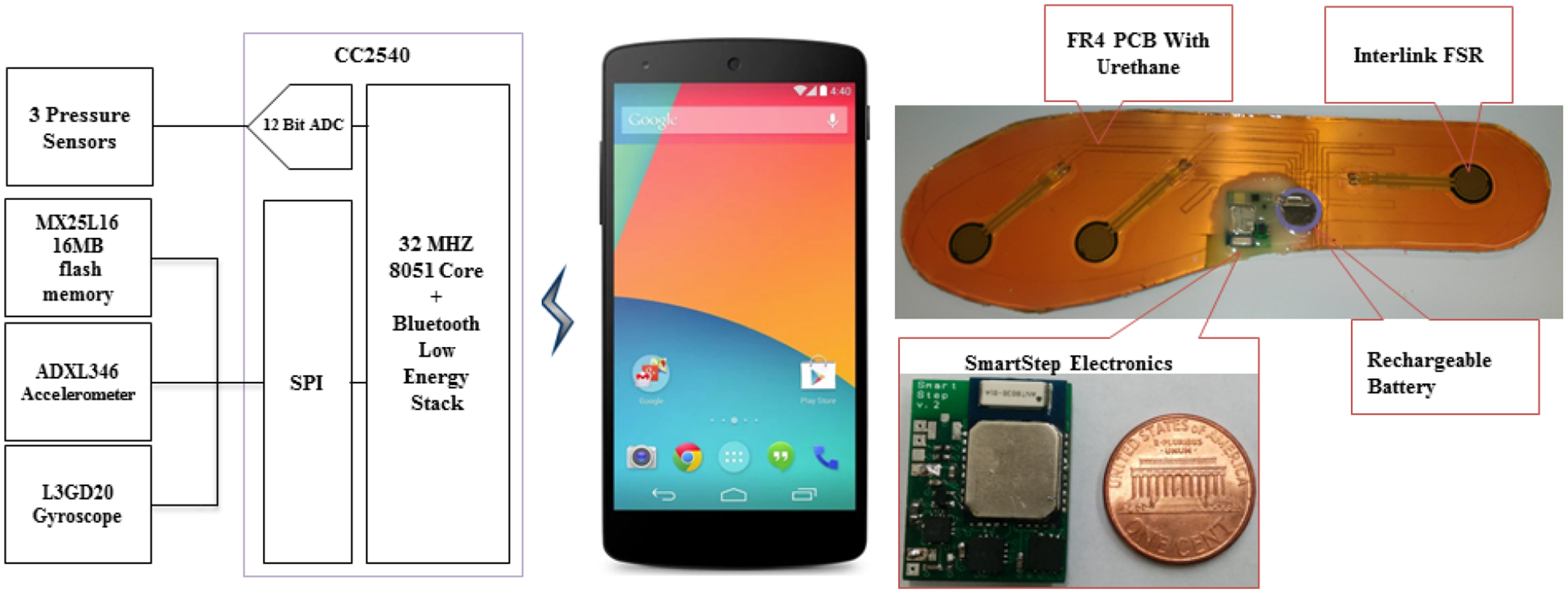
SmartStep block diagram and fully encapsulated sensor-equipped insole.

The SmartStep insole system is assembled on a flexible 8 mil (0.2 mm)-thick FR4 PCB. Three Interlink FSR402 pressure sensors are located under biomechanically important support locations: the heel, the first metatarsal head and the big toe. After placing the pressure sensors at the proper locations, the entire FR4 PCB is laminated to provide higher strength to the assembly. The electronic PCB is mounted on the FR4 PCB at the location of the arch of the foot (where forces developed during ambulation are minimal) and encapsulated in epoxy resin. The whole assembly is then encapsulated in urethane rubber for cushioning and protection. Individuals can wear additional cushioning materials based on the need. The insole weighs 71 g in total. Figure 1 demonstrates the block diagram of SmartStep, along with pictures of the fully mounted PCB and fully encapsulated insole.

### 2.2. SmartStep Firmware

The firmware for the SmartStep is built around TI’s BLE stack version 1.4.0 [29]. The algorithm for the application from the SmartStep point of view is completely reentrant. It is an “always on” system, which has no reset or power switch on-board.

**Figure 2.**
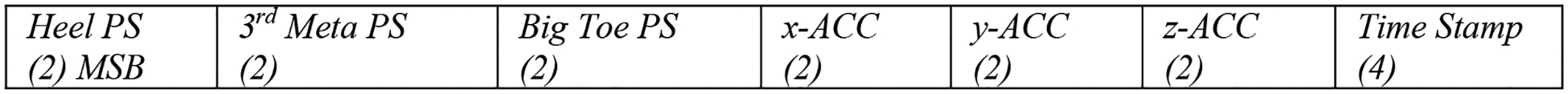
Notification packet when the gyroscope is not used. PS, pressure sensor; ACC, accelerometer; MSB, most significant bit.

Below are the definitions of the terms from the BLE perspective that are repeatedly referred to in the article:

1. Characteristic: A characteristic contains a single readable and/or writable value and descriptors that describe the characteristic’s value. In the case of SmartStep, there is a characteristic that is used for the purpose of control and another for sensor data transmission.
2. Descriptor: A Descriptor is a defined attribute that describes a characteristic value.
3. Service: A service is a collection of characteristics.
4. Server: A device that has defined characteristics in it. In the present context, SmartStep is the server, and the terms are used interchangeably in the article.
5. Client: A device that reads from or writes to the server. The Android application is the client in the case of the SmartStep system, and the terms are used interchangeably in the article.
6. Connection event: The event that takes place periodically when there is an established connection, where the server and the client can communicate the data.
7. Connection interval: The time interval that defines the connection events’ period.
8. Event timers: The timers provided by the BLE stack that are used to schedule events. In SmartStep, one event timer handles the sensor reading event and another timer handles the notification events. Normally, both timers operate at 25 Hz (a sampling frequency shown to be sufficient for accurate posture and activity recognition in an earlier study [21]).
9. Notification: A method of data communication from the server to the client during the connection events, in which the client does not acknowledge the reception of the data from the server. As per the BLE standard, each notification data packet can have a maximum size of 20 bytes. SmartStep transmits sensor data to the smart phone using notifications, each containing sensor data acquired during a sensor read event along with a timestamp. Figure 2 shows the format of a notification packet when pressure sensors and the accelerometer are being read during sensor read events, excluding gyroscope readings (the number of bytes for each field is shown in the brackets). As seen in Figure 2, when the gyroscope is not read, the notification packet contains 16 bytes of data.

**Figure 3.**
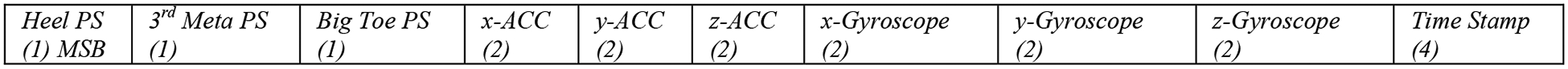
Notification packet when the gyroscope is used.

When the gyroscope is also being read during sensor read events, a notification packet contains 19 bytes of data, as shown in Figure 3:

- Indication: A method of data communication from the server to the client during the connection events, in which the client acknowledges the reception of the data from the server.
- Advertising event: The event that takes place in SmartStep periodically when there is no established connection. During this event, SmartStep transmits the advertising packet, attempting to get connected with the client.
- Advertising interval: The time interval that defines the period of advertising events. In the case of SmartStep, this is 1 s.
- Sleep State: A state of the SmartStep system, while all the electronic components are in sleep mode, drawing minimal current from the battery. During the firmware initialization action in SmartStep, the default connection interval is set to be at 160 ms. Automatic updating of the parameters defining the connection interval can take place on the fly, when the SmartStep intends to have it altered. The SPI module, ADC module, event timers, accelerometer, gyroscope and flash memory are initialized during the initialization sequence, but become active only when they are enabled by the client. Actions from the smart phone’s side will be discussed in the Section 2.5.

In a general usage scenario, the SmartStep advertises periodically, so that any clients scanning for the server can find and try to establish a connection with the SmartStep. Once there is a connection that is established between the SmartStep and the client, the SmartStep waits for the control commands from the client. The client writes a characteristic value, communicating with the SmartStep about the initial setting needed for the particular data logging session. These settings define whether or not the gyroscope is read and whether or not the data retention scheme has to be in place for the session. After this, the client starts the data logging session, and the SmartStep reads the sensors, forms a notification packet of the read data and transmits the data to the client. This mode of operation is maintained until the client commands to stop the data streaming or there is a connection loss. If the client commands to stop the data streaming, the SmartStep still maintains the connection, but halts the sensor readings. At this stage, the client can choose to get disconnected from SmartStep or to start another data logging session. While in the data logging session, if there is an unintended connection loss, the SmartStep may enter a flash data retention scheme or halt the sensor readings based on the initial setting for the current session. If the data retention scheme is active, then SmartStep makes sure that all of the flashed data contents are transmitted once the BLE connection gets reestablished again by entering the modified notification mode.

**Figure 4.**
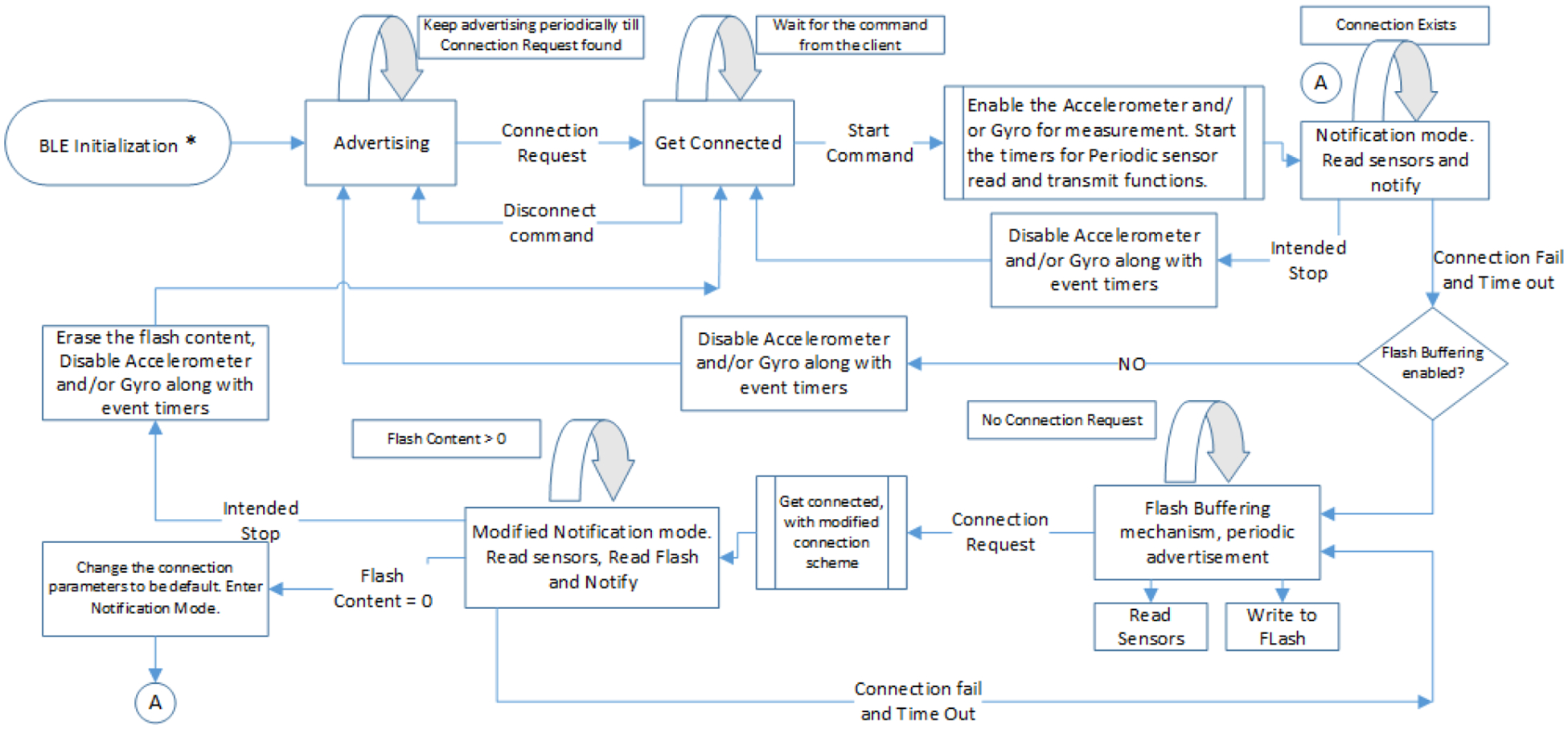
System operation and state flow graph.

The state flow graph in Figure 4 describes the whole operation of the SmartStep, which can be operating in one of the below modes at any given point of time:

- Periodic advertisement mode: SmartStep remains in limited discoverable mode, which means the advertising event takes place periodically and not indefinitely. The advertising period is 30 s (advertising state) followed by another period of 30 s with no advertising (sleep state). There is no connection with the client yet.
- Notification mode: SmartStep enters the notification mode after establishing a connection with the client. The sensors are sampled at 25 Hz, and the connection interval is 160 ms. The SmartStep can be in one of the following states in this mode: sleep state, sensor read event state, notification set event state or BLE connection event state.
- Flash buffering mode: SmartStep enters flash buffering mode when there is a loss in the established connection between SmartStep and the client. In this mode, SmartStep samples sensors at 25 Hz and writes the sensor readings to the flash memory. In an attempt to get reconnected with the client, SmartStep keeps advertising periodically. The SmartStep can be in any of the following states in this mode: sleep state, sensor read event state, advertising event state or flash buffer write event state (which is handled by the notification set event).
- Modified notification mode: When the SmartStep is reconnected with the client from the flash buffering mode, SmartStep enters the modified notification mode. In this mode, the connection interval is 80 ms (half that of the normal notification mode), the notification set event interval is 20 ms and sensors are sampled at 25 Hz. The SmartStep can be in one of the following states in this mode: sleep state, sensor read event state (which handles current sensor data, as well as flash buffered data), notification set event state or BLE connection event state.

### 2.3. Low-Power Schemes

This section describes the system design aspects utilized in SmartStep, which help in achieving low-power functionality in an “always-on” SmartStep system:

1. During advertising, the SmartStep remains in limited discoverable mode.
2. For the sensor data transmission from the server to the client, notification methodology is used as opposed to indication methodology.
3. Enabling/disabling of a notification is carried out in two steps from the client side. In the first step, the client application writes to a characteristic variable in the server, before enabling or disabling the notification. If the action from the client requests for enabling of the notification stream, the accelerometers and/or gyroscope are put into measurement mode (the mode in which the sensors sense and measure the motion/rotation), and the event timers are started. As a second step, notifications are turned on.
4. The event timers are running and sensors are in the measurement mode as long as the SmartStep is in the notification/modified notification mode. Before stopping the notifications, the client again writes to the characteristic variable, after which, the whole system is put in a lower power mode.
5. While in measurement mode, the ADXL346 operates in low power, with the output data rate (ODR) set to 25 Hz. This low-power mode is slightly noisier than the normal operating mode of ADXL346 [30], but consumes 50% less current than in the normal mode.
6. Only during ADC reads, the resistive pressure sensors are supplied with current.
7. BLE allows up to 80 bytes of user data to be transmitted in each connection event. Hence, SmartStep’s connection interval is defined as 4-times that of the notification set events, and this implies, in the place of 4 connection events, one for each notification packet, that SmartStep has one connection event, saving the radio power consumption.

### 2.4. Flash Management Scheme

**Figure 5.**
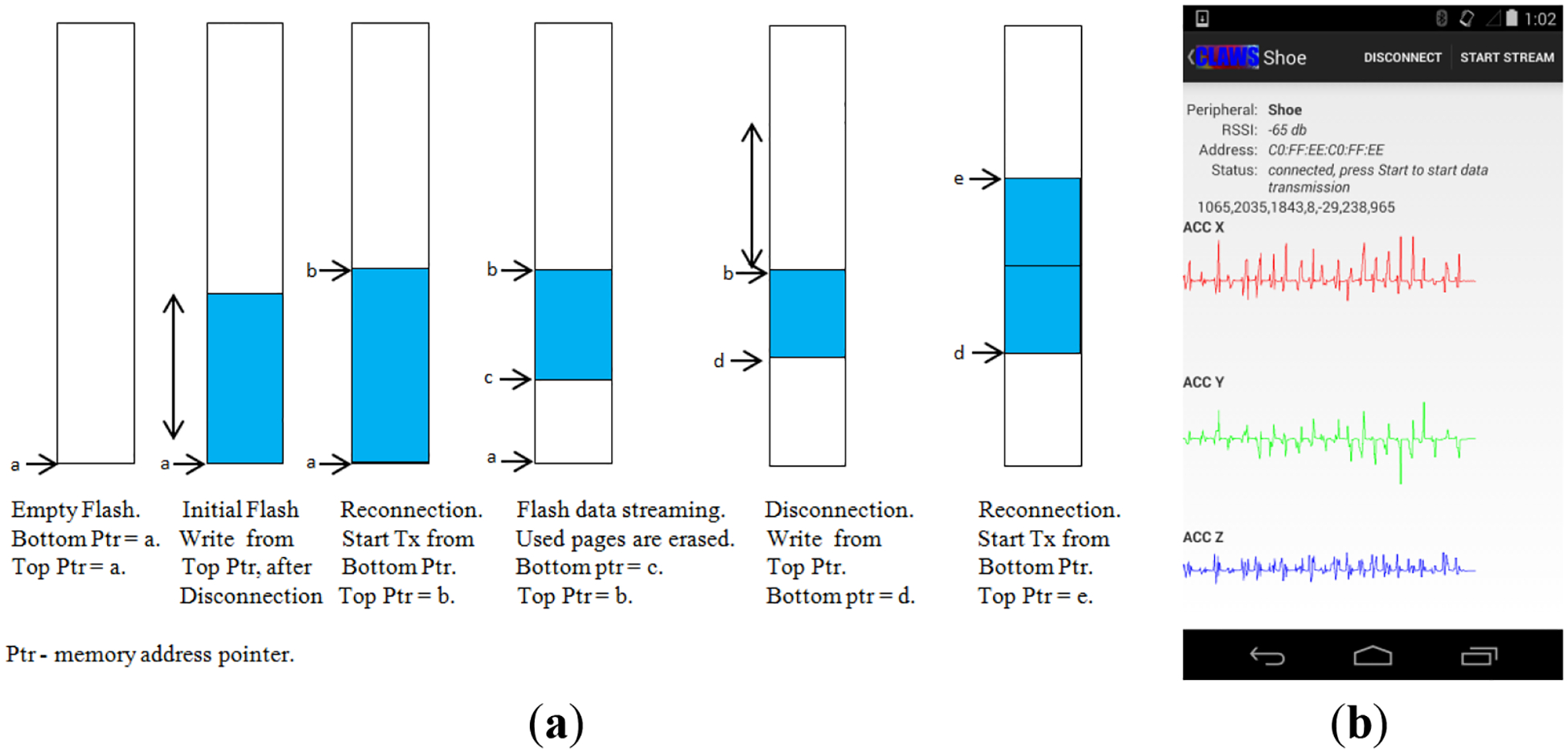
(**a**) Flash management mechanism; (**b**) screenshot of the android app.

Flash memory is utilized to save the sensor data, when there is an unwanted connection loss. This unwanted loss of connection can happen when the wearer of SmartStep walks out of the range of the network (away from his phone), while the SmartStep is in notification or modified notification mode. When any such situation arises, SmartStep ensures that the sensor data is preserved by entering into the flash buffering mode. In this mode, rather than communicating the data over BLE, SmartStep keeps writing the data to the flash memory in pages (256 bytes). Flash buffering mode is automatically switched to modified notification mode when the SmartStep comes in the BLE range of the phone again. In the modified notification mode, the flashed data is read in pages, and this buffered data is notified along with the current sensor readings to the phone. Both the most recent (real-time) data and flash buffered data get transmitted in the case of reconnection after a connection loss, thus avoiding the potential lag due to the transmission of buffered data. Periodic housekeeping happens in the firmware, erasing the read pages in sectors (4 Kb). The Flash memory has the capacity of 16 M bit, and given that the sensors are read at 25 Hz and each notification packet is 16 bytes in width (in case there is no gyroscope reading), the flash memory can be used to buffer the data for a maximum of 1.5 h. If the flash memory gets completely filled, then the sensor reading is halted until the SmartStep connects with the smart phone again. Figure 5a explains the operation of the flash buffering mechanism.

### 2.5. Android Implementation

The current implementation of the Android application (App) is based on Google’s most recent Android version 4.4.2, which has native android support for BLE [31]. The app can scan for available SmartStep servers, connect to the server, search for the available services from the server, read/write characteristic variables in the server and enable/disable notifications from the server. The app user can select whether the data retention mechanism is needed for the data logging session and also whether the SmartStep needs to read the gyroscope or not. During the session, the sensor data notified by the SmartStep can be displayed on the screen in real time and also can be written to a comma separated value (csv) file on the smart phone’s storage. During the data logging session, if there is an accidental connection loss, the app immediately gets connected back in the same mode as it was operating. If a connected server goes outside the range and if there is a time out, the app starts periodic scanning, in an attempt to find the server. When the server comes back in the BLE range, the app reconnects with the server and re-enables the notification stream.

When there is inconsistent network strength, reconnecting to the server is prone to constant disconnections, which may cause additional data loss in SmartStep. To avoid this from occurring, the application checks radio signal strength indicator (RSSI) during scanning and automatic reconnection takes place only when RSSI is higher than −75 dB, which is an empirically established threshold determined by the authors. Figure 5b shows a screen shot of the application while it is capturing a subject’s walk, while wearing SmartStep.

## 3. Experimental Section

### 3.1. Data Retention Scheme Tests

#### 3.1.1. Formal Test of Data Retention Scheme for Single Disconnection

A 20-min experiment was conducted to test the functionality of the data retention scheme and frequency scaling for a single disconnection. The SmartStep was in connection with the smart phone app initially for a minute, after which the wearer went outside the range of BLE network (typically 50 m) for 5 min. At that time, the sensor data were logged into the flash memory, and once the wearer came back, the app automatically connected with the SmartStep. Following this, the SmartStep sent the flash buffered data along with current sensor readings. The entire experiment ran for 20 min, and the received data from the experiment were stored in a csv file from the app. The presence of time stamps in the notification packets helped in sorting the received data, and a quantitative measure on data received (DR) and data loss (DL) was calculated using a MATLAB script as:

(1) $$DR=\left(\frac{NTS}{LTS-FTS+1}\right)\times 100$$

(2) $$DL=\left(1-\frac{NTS}{LTS-FTS+1}\right)\times 100$$

where NTS is the total number of timestamps received, LTS is the last timestamp received and FTS is the first timestamp received.

#### 3.1.2. Formal Test of Data Retention Scheme for Multiple Disconnections

An hour-long experiment, emulating multiple nested disconnects, was conducted. First, the SmartStep was in connection with the app for 10 min. Following this, the wearer went outside the network range for 10 min. When the wearer came back in range, as per the connection scheme, it would take 10 min to get all of the flashed data; however, the wearer went out of the network after 5 min of the first reconnection and came back into the network 5 min later. Following this, the wearer was in the network for the next 30 min. This is a very common usage scenario in which wearers of SmartStep could move in and out of the vicinity of the phone arbitrarily. A quantitative measure on DR and DL during the whole experiment session was calculated using the MATLAB script, as done before in Test 3.1.1.

### 3.2. Power Consumption Tests

Multiple power consumption tests were conducted to measure the power consumption and to estimate the expected battery life of the SmartStep in different modes of operation. The power consumption by the SmartStep depends on the types of sensors being read and the state of the BLE stack. A 10-Ω resistor was placed in series with the battery supply, and the voltage across the resistor was captured using an oscilloscope. The average current was calculated in the method explained in [32]. To find conclusive figures on power consumption *vs*. functionality trade off, all the possible modes of SmartStep were considered, namely:

- Periodic advertisement mode.
- Notification mode:
  1. With pressure sensors and accelerometer.
  2. With pressure sensors, accelerometer and gyroscope.
- Flash buffering mode.
- Modified notification mode.

In addition, the power consumption breakdown between different states of the system for a given mode of operation was calculated by considering all of the different states in which the system can be operating, at any given time, in the given mode of operation. Each individual state’s contribution towards the total power consumption for a given particular mode was calculated. Equation (3) gives a quantitative measure of the contribution of a given event state in notification mode (*C*_XX_):

(3) $$C_{XX}=\left(\frac{A_{XX}}{ANM}\right)\times 100$$

where *A*_XX_ is the average current consumed by the state XX and ANM is the average current consumption in notification mode.

### 3.3. Test for Functionality and Reliability of the Insole Monitor

#### 3.3.1. Mechanical Reliability for a Prolonged Wear

The characteristics and response of the pressure sensors after machine-generated cyclic loading experiments were reported in a previous study [28]. However, during dynamic human activities, the insole will be subjected to bending (which cannot be emulated with the available machine test frame), and this can affect the sensor behavior. Hence, to understand the behavior of the pressure sensors in the SmartStep over prolonged human wear, the following experiment was conducted.

The responses of the pressure sensors at the heel and meta positions were recorded before the first wear with the help of a calibrated weight (36 Kg). The insole was worn by one healthy subject, with the shoe size equal to the size of the manufactured SmartStep insole (U.S. M9) (for different shoe sizes, there will be different insoles, which are all based on a generic design). The subject was wearing the insole in free living conditions and performing activities that included, but were not limited to, walking, running, bicycling, *etc*., as per his daily schedule. Every week, the response of the pressure sensors were recorded using the same calibrated weight. The test ran for a total of 4 weeks.

#### 3.3.2. Real-Life Experiment to Understand the Achievable Battery Life on a Single Charge

Power consumption tests conducted in Section 3.2 gave an estimate of the expected battery life while using SmartStep, in different possible modes of operation, when considered individually. However, in real life, the actual power consumption by SmartStep depends on a mixture of different modes in which the SmartStep can be operating at different times. Hence, to get an understanding of SmartStep’s battery life in a real-life use case, an experiment was conducted on a single healthy subject wearing SmartStep, and the sensor data was logged in the smart phone over BLE.

Before starting the experiment, the SmartStep battery was fully charged to 3 V. The data was logged continuously until the battery depletion. During the experiment, the subject was in free living conditions and was performing various activities, which were not monitored. After the experiment, the logged data were processed, and using Equations (1) and (2), a quantitative measure on DR and DL was calculated. The timestamps in the received data were also used to get a conclusive figure on the achievable battery life, in a real-life use case.

## 4. Results

### 4.1. Data Retention Scheme Tests

#### 4.1.1. Formal Test of Data Retention Scheme for Single Disconnection

**Figure 6.**
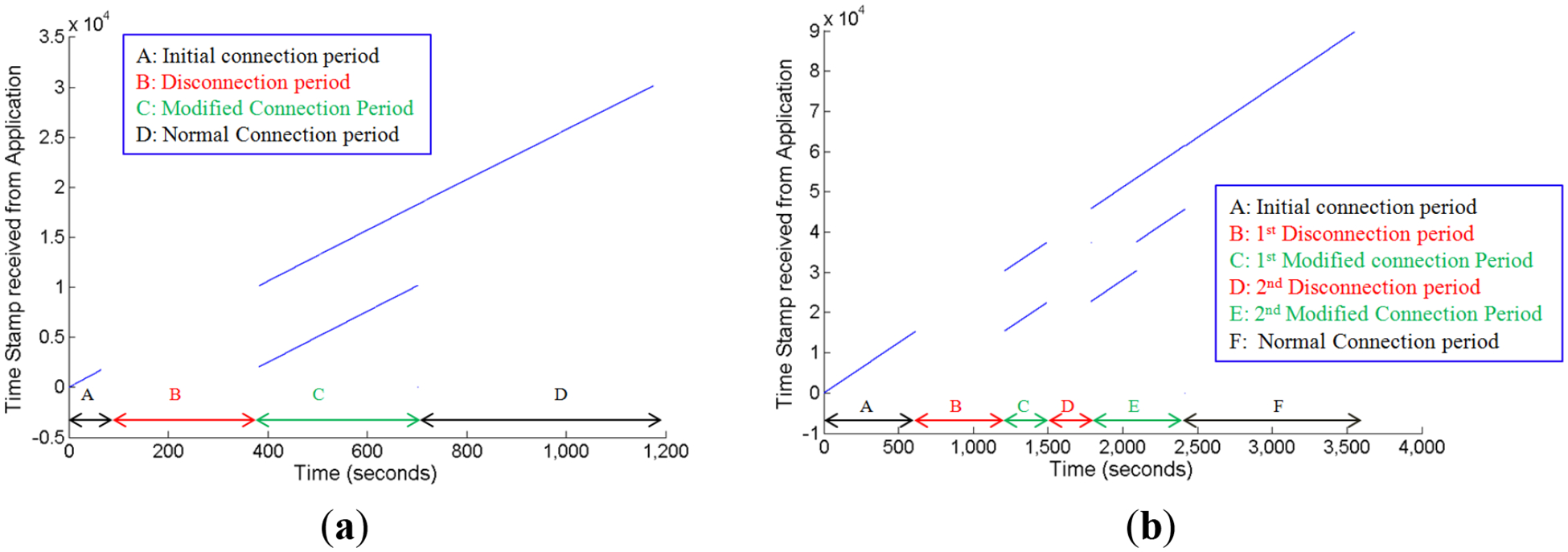
Scatter plots for the formal test of the data retention scheme: (**a**) single disconnection; (**b**) multiple disconnections.

Figure 6a shows the scatter plot of the timestamps received for the data retention experiment. In the figure, during time Intervals A and D, the SmartStep was in normal notification mode; Interval B is the disconnection period, and during Interval C, SmartStep was in modified notification mode. In the Interval C, the bottom blue line represents timestamps for the flash buffered data, and the top blue line represents the timestamps for the data read in real time, indicating that both the buffered and the real-time data are being transmitted. During this 20-min test, 97.5% of the sensor data was received successfully.

#### 4.1.2. Formal Test of Data Retention Scheme for Multiple Disconnections

Figure 6b shows the scatter plot of the timestamps received for the experiment with multiple nested disconnects. In the figure, Intervals A and F correspond to the normal notification mode, Interval C is the first modified notification interval, Interval E is the second modified notification interval and Intervals B and D are the first and second disconnection intervals, respectively. In the Intervals C and E, the bottom blue lines represent timestamps for the flash buffered data and the top blue lines represent the timestamps for the data read in real time. In the experiment period of one hour, 98.9% of the sensor data was received successfully.

### 4.2. Power Consumption Tests

**Figure 7.**
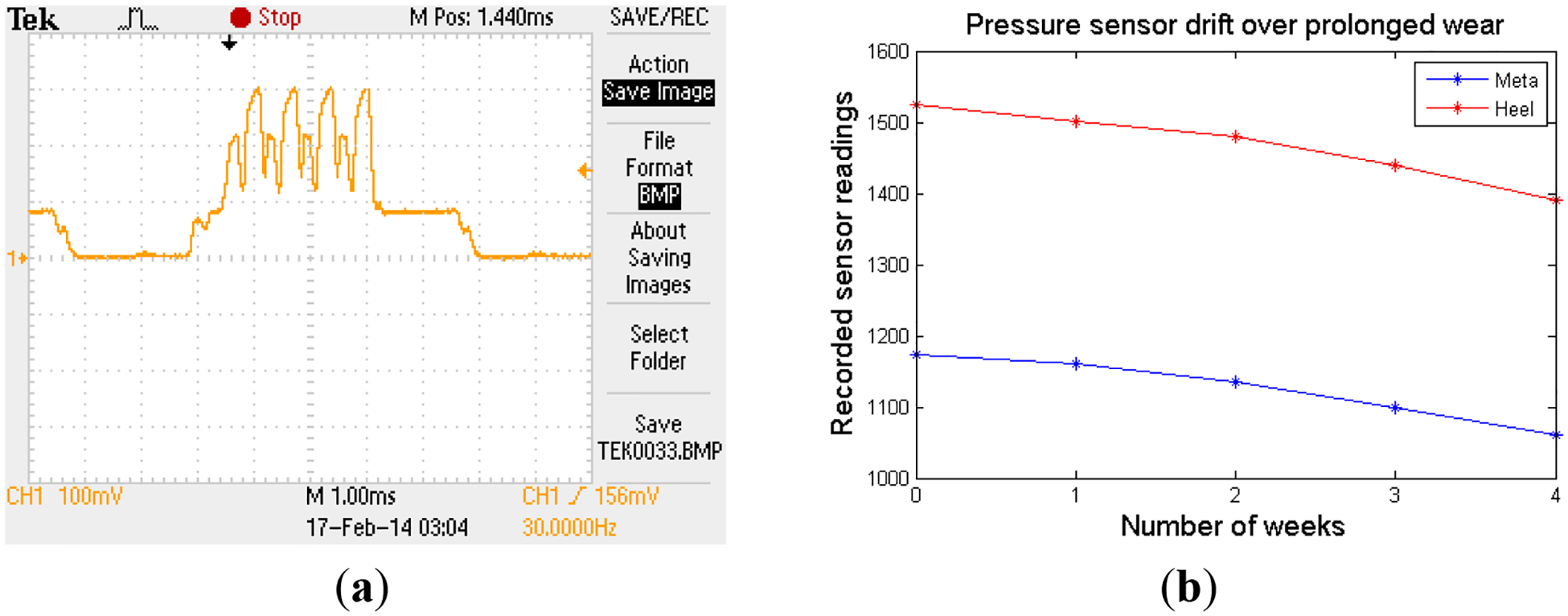
(**a**) Scope trace for connection event; (**b**) pressure sensor readings plot displaying the drift.

Figure 7a shows the scope traces captured for the connection event. It can be seen from the trace that there were four transmissions of notification packets happening at the connection event, confirming the notification packet grouping methodology used. Similar to the traces in Figure 7a, scope traces for all of the different states of activities of SmartStep in different modes were captured, and the average current consumption along with the expected battery life were calculated. The results (rounded to the nearest hundredth) are presented in Table 1:

Table 2 presents the power consumption breakdown among the possible states for the notification mode, with and without the gyroscope.

### 4.3. Test for Functionality and Reliability of the Insole Monitor

#### 4.3.1. Mechanical Reliability for Prolonged Wear

Figure 7b shows the graph of pressure sensor readings plotted against the time of wear. The meta-sensor drifted a total of 10% over the span of four weeks of wear, while the heel sensor drifted 9% in the same period.

#### 4.3.2. Real-Life Experiment to Understand the Achievable Battery Life on a Single Charge

The SmartStep was able to continuously send sensor data to the smart phone for 24.6 h over BLE on a single charge. There were two disconnections that happened during the experiment, in which the SmartStep was able to preserve the sensor data. In this experiment, 98.6% of the sensor data were received successfully.

## 5. Discussion

A low-power, fully integrated insole monitor—SmartStep—was designed and developed in this study. This insole monitoring system was designed with pressure sensors, an accelerometer, a gyroscope, BLE connectivity and flash memory along with a rechargeable battery, which were integrated in the insole. The insole system was aimed at resolving the limitations of the existing shoe monitoring systems.

From the formal experiments on the functionality of the data retention scheme, it was confirmed that the implementation of the flash buffering scheme was able to preserve the sensor data, whenever there was a loss of connection. The results for these experiments showed that the data retention scheme in SmartStep could handle multiple disconnection/reconnection events successfully and retrieve a very high percentage of data (~99% during a one-hour experiment). A portion of the lost data (~1%) can be accounted for by the time difference between the connection loss and time out period (6 s in the case of SmartStep), only after which, the flash buffering mechanism gets activated, and the sensor data sampled during the timeout period are not buffered in the flash. Another reason can be that, as the wearer moves away from the smart phone, the BLE signal strength gets gradually reduced and not all the packets that are sent are received. The design choice of implementing the notification methodology as a means of data communication, in which there is no acknowledgement for the received data from the client to the server, can be contributing to the packet loss. However, choosing an indication method over a notification method would have affected the power consumption figures, by increasing the current consumption by BLE connection events.

Among all of the modes tested for power consumption, the periodic advertisement had the least average current consumption, as the sensors were inactive in that mode and the radio was on for a short period during advertising events. The flash buffering mode was the next least in power consumption, since, in this mode, SmartStep read the sensors, wrote the sensors’ data to flash memory and did not have an active BLE connection. Power consumption during the notification mode was higher than the flash buffering mode, as in this mode, the sensors were read and, also, the radio was active because of the active BLE connection. The modified notification mode was the highest power consuming amongst all, as in that particular mode, the BLE connection frequency was doubled to that of the normal notification mode. During the normal notification mode (without gyroscope), the average current consumption was 1.45 mA, and this is a significant improvement (~28 times) from that of the SmartShoes with a 40 mA average current consumption.

Since the pressure sensors were placed right under the foot inside the shoe, which was quite a harsh environment in free living conditions, we expected that these sensors would wear out and also drift over time. It was encouraging to know that the pressure sensors in SmartStep were able to tolerate prolonged human wear in free living conditions. This result indicates that, from the mechanical reliability aspect, the SmartStep can be used in prolonged human subject studies lasting a month; however, for studies longer than a month, it will be nicer to have a more reliable system, with less drift, which will be emphasized in future research.

SmartStep had a battery life of 24.6 h on a single charge. This battery life figure falls between the characterized operating hours for the notification mode and modified notification mode, in which the SmartStep could be operating in real life. This shows that the SmartStep, indeed, is a low-power system. In terms of battery life, with a 500 mAh battery capacity, SmartShoes could operate for 12 h; with a 2,000 mAh battery [5], it lasts for 20 h; while with a 40 mAh battery capacity, SmartStep could operate for more than 24 h. Considering 12 hours of wear per day, SmartStep could be used for more than two days on a single charge, providing a battery life suitable for the use of SmartStep in research studies.

Table 2 results indicate that more than 50% of the total power consumption in the notification mode was because of BLE activities (which include “notification set event” and “BLE connection event”). Hence, the battery life can be improved by reducing the number of “BLE connection events” and “notification set events” in the system, which will be dealt with in future research. By incorporating a higher capacity coin cell battery in the SmartStep assembly, such as [33] with a 100 mAh capacity, SmartStep’s battery life can be doubled. There is a plan to move to a CC2541-based BLE module, which is a lower power solution compared to CC2540. Using CC2541 along with a DC-DC convertor would save more than 30% of the power consumption, when the radio is active [34]. Furthermore, there is an ultra-low power variant to the ADXL346 accelerometer, ADXL362 [35], which consumes ~20-times less current than ADXL346. However, even after these improvements, the SmartStep can be used for 7–8 days only, on a single charge, and has to be recharged after that. Implementing a simple recharging mechanism (such as “wireless charging” as opposed to “wired charging” in the present implementation) will also be the topic of future research.

## 6. Conclusions

This article described the solutions to the system design challenges identified during the development of SmartStep—a fully integrated, low-power insole monitor. The conducted power tests demonstrated that average power consumption in SmartStep was ~28-times lower than that of the original SmartShoe monitor, and the real-life experiment conducted in this study suggested that the present implementation of SmartStep can be used for more than two days on a single charge. The present implementation of the data retention scheme was functioning as it was intended and with minimal loss of data (<1.5%). The results from the mechanical reliability experiment suggested that the SmartStep pressure sensors tolerated prolonged human wear. These results, along with the proposed improvements, suggest that the SmartStep can be used in the long-term monitoring of gait, physical activity or other parameters. Utilizing the SmartStep monitoring systems in studies on the physical activity and gait monitoring of human subjects, in free living conditions, and processing the sensor data captured from SmartStep will be the topics of our future research.

## Figures and Tables

**Table 1.** Power test results, average current consumption and expected battery life for different modes.

Scheme Used	Average Current Consumption (mA)		Expected Battery Life (Hours)	
	Without Gyro	With Gyro	Without Gyro	With Gyro
Periodic advertisement mode	0.05	0.05	900.00	900.00
Notification mode	1.45	1.65	31.03	27.27
Flash Buffering mode	1.00	1.20	45.00	37.50
Modified Notification mode	1.99	2.19	22.61	20.54

**Table 2.** Power consumption breakdown in the notification mode.

Event State	Notification Mode (Without Gyro)		Notification Mode (With Gyro)	
	Average Current Consumption (mA)	Contribution (%)	Average Current Consumption (mA)	Contribution (%)
Sleep state	0.11	7.65	0.11	6.73
Sensor read	0.44	30.00	0.61	36.38
Notification Set	0.36	25.18	0.39	24.23
BLE connection	0.54	37.17	0.54	32.66
Total	1.45	100	1.65	100
